# Editorial: Follicle-Stimulating Hormone: Fertility and Beyond

**DOI:** 10.3389/fendo.2019.00610

**Published:** 2019-09-06

**Authors:** Manuela Simoni, Ilpo Huhtaniemi, Daniele Santi, Livio Casarini

**Affiliations:** ^1^Unit of Endocrinology, Department of Biomedical, Metabolic and Neural Sciences, University of Modena and Reggio Emilia, Modena, Italy; ^2^Department of Surgery and Cancer, Institute of Reproductive and Developmental Biology, Imperial College London, London, United Kingdom

**Keywords:** FSH (Follicle Stimulating Hormone), fertility, FSHR (Follicle-Stimulating Hormone Receptor), polymorphism, gonadotropins

Propagating life to the next generation is a hormone-dependent process relying on the individual wish to generate own progeny and resulting in maintenance of species. This Research Topic is dedicated to Follicle–Stimulating Hormone (FSH) and its receptor (FSHR) and their role in reproduction.

FSH is a typical example of a drug which entered clinical use in the “pre-evidence-based medicine era,” just for its efficacy in stimulating gonadal function and fertility in hypogonadotropic hypogonadism. More recently, FSH entered clinical use in controlled ovarian stimulation in order to obtain multiple follicular growth for assisted reproduction. Given the progressive increase in couple infertility, the demand for assisted reproduction grows steadily and the FSH market is flourishing. Yet, very little was known about the FSH mode of action until a few years ago, and the therapeutic use of FSH is still far from being evidence-based. But great progress in our understanding of FSH action was made in the last two decades and, since not many scientists around the world are active in the gonadotropin/FSH research “niche,” we thought it was time to call them to report to tell us their view on the state-of-the-art. The result is this “Research Topic.”

Starting from the cover image, the illustration depicts “San Giuseppe con il Bambino” (Saint Joseph with his child son) by Gian Lorenzo Bernini (1598–1680), an almost unknown work by the great Italian renaissance artist painted using the “sanguigna” technique on the left wall of the private Chapel in Palazzo Chigi, Ariccia, near Rome. We chose this picture for its evocative power: a seemingly old man holds his sleeping son in his arms, looking at him in adoration. This picture reminds us of several actual aspects of reproduction: late paternity, fertility/infertility, perhaps even “assisted” reproduction, and the centrality of parenthood for life. All aspects related to FSH. We would like to thank architect Francesco Petrucci, the curator of the museum, for granting us permission to use Bernini's work for this issue.

We asked the authors who participated in this Research Topic to consider the following questions: Why is FSH absolutely necessary for fertility in some species and not in others? How does FSH interact with other hormone-receptor systems (e.g., -but not exclusively-Luteinizing Hormone and its receptor) in the cells where the FSHR is expressed? How do these interactions contribute to the apparent pleotropic and sometimes even redundant interplay between the LH and FSH? What is the current knowledge about FSH-mediated signaling and FSHR structure-functions? Is FSH helpful to increase fertility potential in infertile couples? Can we effectively base novel therapeutic approaches to infertility on genetic variants of FSH and its receptor (pharmacogenetics)? Can we develop low molecular weight, selective FSH analogs stimulating/modulating specific effects? Is it possible to block some specific action/mechanism of FSH for contraceptive purposes? Does FSH have a role in cancer and other diseases? The result of this call is a very comprehensive collection of 21 articles dealing with such aspects.

## Structure-function relationships of FSH and FSHR

Ulloa-Aguirre et al. reviewed the seminal features of structure and function of the follicle-stimulating hormone receptor (FSHR). The roles of the different functional domains of FSHR, i.e., extracellular, hinge, transmembrane, and intracellular, are described in ligand binding, signal transmission, receptor trafficking and dimer/oligomerization.

FSH bioactivity is influenced by glycosylation. Two articles, by Bousfield et al. and by, Campo et al. respectively, reviewed the current knowledge about the naturally occurring FSH isoforms and the endocrine control thereof. FSH is really a fascinating molecule, and, while the biochemistry of glycosylation is relatively well-understood, its pathophysiological consequences require more investigation.

Riccetti et al. provided an experimental study comparing extensively the biochemical and signal transduction properties of the reference follitropin alfa (recombinant FSH) and two common biosimilars. The paper demonstrates that the tested preparations have some differences in glycosylation, as expected, but substantially similar signal transduction properties, at least at physiological concentrations (Riccetti et al.).

## Mechanism of Action of FSH

The molecular mechanism of action of FSH is reviewed by Casarini and Crepieux, who give a very comprehensive and updated view of the complexity of FSH action, much beyond the well-known cAMP- mediated signaling system (Casarini and Crepieux). It is now clear that FSH may act as a pro-apoptotic factor in steroidogenic cells, a new aspect of FSH action probably fundamental to govern follicular atresia.

Landomiel et al. have made an extensive discussion on the G protein-coupled receptor (GPCRs) structure and mechanism of activation, with particular attention to the efficacy in activating or inhibiting selective signaling pathways. They deeply developed these novel concepts in the understanding of the FSH-FSHR biology, opening to new classes of pharmacological tools able to bias FSHR signaling.

Gonadotropin receptors act as dimers/oligomers and there is evidence that the FSHR can heterodimerize with the LH/CG receptor in the cells where the two receptors are co-expressed (granulosa cells). This new aspect of FSH function is analyzed in the contribution by Szymanska et al., demonstrating how this characteristic can amplify/diversify the action of FSH, via biased signaling.

Sayers and Hanyaloglu provided an update on how gonadotropin hormone receptor activity is organized in intracellular compartments. This review comprehensively elucidated novel aspects on the role of cellular trafficking machinery and spatial organization of GPCR signaling in regulating physiological functions.

## Physiological and Therapeutic Effects of FSH

The role of FSH in spermatogenesis has been a matter of debate for many years. Depending on the experimental model, the FSH role ranges from absolutely necessary to dispensable for fertility in the male.

Oduwole et al. review the experimental evidence of the role of androgens and FSH for spermatogenesis in mice. It appears that a strong FSH stimulus is able to support spermatogenesis even in the absence of androgens. While a certain degree of redundancy between FSH and LH action on spermatogenesis emerges, these findings suggest a possible role of high-dose FSH in treatment of spermatogenic failure.

Another approach to study FSH action is based on gain-of-function mouse models. McDonald et al., along with others, have generated genetically modified mouse models with gain-of-function mutations in FSH and its receptor. In this article, the existing mouse models for activation of FSH action are discussed, and additional novel genetic models are proposed to refine the information on FSH action.

FSH is important for quantitatively and qualitatively normal spermatogenesis. Muratori and Baldi describe the potential role of sperm DNA fragmentation (sDF) index in reproductive medicine, focusing on the beneficial effect of FSH administration on this parameter.

## Genetics of FSH Action and Pharmacogenetics

Laven reviewed the current evidence regarding the role of gonadotropin-related single nucleotide polymorphisms (SNPs) in polycystic ovarian syndrome (PCOS), suggesting that *FSHR, FSHB*, and *LHCGR* SNPs could influence the PCOS phenotype and the response to FSH administration.

In their review Conforti et al. dealt with the question whether the clinical use of FSH in women, essentially in assisted reproduction, could benefit from a pharmacogenetic approach. They concluded that a pharmacogenomic approach to ovarian stimulation could become a clinical reality in the future, with specific variants of FSH Beta and FSHR being promising genetic markers to better standardize controlled ovarian stimulation in women undergoing ovarian stimulation.

Schubert et al. reviewed the literature dealing with a FSH pharmacogenetic approach to male idiopathic infertility. They show that the current evidence is not univocal and propose a possible study design for future clinical trials for the pharmacogenetic use of FSH in male infertility.

## FSH and FSH Analogs for Therapy

An historical overview about development of gonadotropins in general and FSH in particular could not be missing in this issue. We asked the pioneer of gonadotropin isolation and clinical use, Bruno Lunenfeld to contribute such a review (Lunenfeld et al.). The reader will find in this article the history of gonadotropins, demonstrating the long path that was required for the development of these drugs.

The position of FSH for therapy of male infertility was examined in the contribution of Behre, who reviewed critically the literature reporting use of FSH of idiopathic male infertility, concluding that the issue is still in need of further research, especially considering the previous attempts using a pharmacogenetic approach.

In his overview about the use of FSH in women undergoing ovarian stimulation for assisted reproduction, Broekmans challenges the view, popular among gynecologists, that the use of FSH needs to be personalized depending on ovarian reserve and response. He concludes that a standard dose of FSH can be used, with the option of triggering final oocyte maturation using a GnRH agonist, avoiding in this way the risk of ovarian hyperstimulation.

Alternative, non FSH-based approaches to modulate the activity of the FSHR have been developed. Kara et al. reported about the possibility to control gonadotropin activity by modulating antibodies. Antibodies were demonstrated to be useful to understand gonadotropin actions and, in other fields, they have shown their utility as therapeutics, e.g., in cancer (Kara et al.). The potential therapeutic applications of antibodies modulating FSH action were reviewed.

A disadvantage of FSH is that is needs to be injected daily. Orally active, small peptide agonist and antagonists have been developed to mimic FSH action. Anderson et al. discuss the major chemical classes of these molecules targeting the FSH receptor, documenting their activity profiles, current status of development, and potential future clinical applications.

## Extragonadal effects of FSH

The issue of extragonadal effects of FSH is quite controversial and is dealt with by two articles. Lizneva et al. provided support to the extragonadal action of FSH with a review summarizing recent studies showing how elevated serum FSH may play a significant role in the evolution of bone loss and obesity, as well as contributing to cardiovascular and cancer risk.

This view is strongly counter-argumented in the article by Chrusciel et al., in which a critical analysis of the results presenting extragonadal expression of FSHR and FSH action, is presented. The authors support the need for the validation of the extragonadal actions of FSH using additional, more accurate, and sensitive supplemental methods, including *in vivo* models and proper positive and negative controls.

## Concluding Remarks

Closing this Research Topic, we sincerely thank all contributors in the hope that the readers will find some answers to their questions and lots of inspiration for new research on FSH/FSHR. While the use of FSH in women is a well-established practice, FSH therapy for male idiopathic infertility remains controversial. Coupling the current knowledge deriving form *in vitro* and animal studies to the genetics of FSH action may pave the way for novel approaches to both increase and inhibit fertility, e.g., for contraceptive purposes. The issue of extragonadal effects of FSH, while fascinating, remains highly controversial.

## Author Contributions

MS, IH, DS, and LC collaborated editing manuscripts published in the special issue.

### Conflict of Interest Statement

The authors declare that the research was conducted in the absence of any commercial or financial relationships that could be construed as a potential conflict of interest.

